# Increased expression of Siglec-9 in chronic obstructive pulmonary disease

**DOI:** 10.1038/s41598-017-09120-5

**Published:** 2017-08-31

**Authors:** Zhilin Zeng, Miao Li, Meijia Wang, Xiaomei Wu, Qinghai Li, Qin Ning, Jianping Zhao, Yongjian Xu, Jungang Xie

**Affiliations:** 1 Department of Respiratory and Critical Care Medicine, National Clinical Research Center of Respiratory Disease, Tongji Hospital, Tongji Medical College, Huazhong University of Science and Technology, Wuhan, 430030 China; 2 Department of Infectious Disease, Institute of Infectious Disease, Tongji Hospital of Tongji Medical College, Huazhong University of Science and Technology, Wuhan, 430030 China

## Abstract

Chronic obstructive pulmonary disease (COPD) is a common inflammatory lung disease. Sialic acid-binding immunoglobulin-type lectins 9 (Siglec-9) is predominantly expressed on innate immune cells and has been shown to exert regulatory effect on immune cells through glycan recognition. Soluble Siglec-9 (sSiglec-9), the extracellular region of Siglec-9, might fulfill its function partly by competitive inhibiting siglec-9 binding to its ligands; however, the role of Siglec-9 and sSiglec-9 in the pathogenesis COPD remain largely unknown. In this study, we showed that Siglec-9 expression in alveolar and peripheral blood neutrophil were increased in COPD patients by immunofluorescence and flow cytometry, respectively. Plasma levels of sSiglelc-9 were elevated in COPD patients by ELISA. *In vitro*, Siglec-9 expression and/or sSiglelc-9 levels were up-regulated by cigarette smoke extract (CSE), lipopolysaccharide (LPS), some cytokines, and dexamethasone (DEX). Recombinant sSiglce-9 increased oxidative burst in neutrophil and enhanced neutrophil chemotaxis toward IL-8 independent on CXCR1 and CXCR2 expression, but it did not affect neutrophil apoptosis or secretions of inflammatory cytokines. In conclusion, Siglec-9 was complementarily increased to induce a negative feedback loop to limit neutrophil activation in COPD, sSiglce-9 enhanced neutrophil ROS and chemotaxis toward IL-8 likely via competitively inhibiting ligands binding to Siglec-9.

## Introduction

Chronic obstructive pulmonary disease (COPD) is characterized by progressive and incomplete reversible expiratory airflow obstruction. Recent clinical surveys have revealed that COPD accounts for 1.6% of all hospital admissions in China and is ranked fourth as a leading cause of mortality in urban areas and third in rural areas of China^1^. Cigarette smoke (CS) and pathogen infection are contributors to the high incidence of COPD^1, 2^. Several lines of evidence support neutrophil as the key effector cells in COPD^3, 4^. Accumulations of neutrophil are frequently found in the bronchoalveolar lavage fluid (BALF) of COPD patients^5^. Abnormal functions of neutrophil are associated with tissue damage, disordered tissue repair, and increased inflammation^6, 7^.

Sialic acid-binding immunoglobulin-type lectins (Siglecs) are characterized by N-terminal domains that bind to sialylated glycans to trigger signals that inhibit or activate inflammation^8^. Siglecs contain CD33 and Siglecs-5-11 in human, but only Siglec-14/5 and Siglec-9 are expressed on neutrophil^9^. By recognizing ligands through interactions with sialic acid residues on cells, Siglec-9/Siglec-E inhibits both immune responses^8^. Numerous studies have reported that Siglecs-9/Siglec-E is involved in modulating neutrophil functions, including induction of apoptosis^10^, inhibition of cellular activation^11^, suppression of migration^12, 13^, modulation of oxidative stress^12, 14^, and regulation of inflammatory cytokines secretion^13^, but their natural ligands have not been fully determined. Neutrophil Siglec-9 is involved in septic shock and rheumatoid arthritis patients^10^. Recent studies have suggested that imbalance of Siglec-5/14 expression contributed to the inflammatory mechanisms in COPD^15, 16^. While we were doing this study, ISHII reported that the SNP of *SIGLEC9* is associated with COPD exacerbation frequency and emphysema^17^. However, the role of Siglec-9 in the development of COPD has not yet been clearly elucidated.

Certain pathogenic bacteria and tumor cells inhibit the immune response by decorating themselves with sialic acids, which could engage Siglec-9 on cells, thereby evading immunosurveillance^18, 19^. Soluble Siglec-9 (sSiglec-9), the extracellular region of Siglec-9, can prevent down-regulation of the immune responsiveness of neutrophil and provide an antibacterial benefit against Group B *Streptococcus* infection likely through competitively inhibiting binding of capsular polysaccharide to Siglec-9^20^. Tomioka *et al*. reported that sSiglec-9 exerts an antitumor function against Mucin1-expressing tumor in mice by avoiding Mucin1 binding to immune cell Siglec-9 to produce the negative immunomodulation and/or by inactivating tumor-associated Mucin1 signaling^21^.

We hypothesized that sSiglce-9 might contribute to neutrophil dysfunction in the development of COPD via competitive inhibition of Siglec-9 ligations binding to Siglec-9. Therefore, the current study was conducted to determine Siglec-9 expression on neutrophil surfaces and sSiglce-9 levels in plasma from smokers with and without COPD. In addition, we evaluated the effect of CSE, LPS, pro-inflammatory cytokines and DEX on neutrophil surface Siglec-9 expression and on sSiglce-9 levels in culture supernatant *in vitro*. Recombinant sSiglce-9 was applied to explore its role in regulating neutrophil function. The impact of sSiglce-9 on HBE inflammatory secretion with or without CSE and LPS was also investigated.

## Results

### Subject characteristics

Clinical characteristics of two group subjects providing for peripheral blood or BALF were shown in Supplementary Tables S1 and S2, respectively. In both of them, the average age and smoking history of COPD patients were similar to those of controls. Compared with COPD patients, controls exhibited significantly higher levels of FEV1 (*p* < 0.05), FEV1% of predicted (*p* < 0.01), and FEV1/FVC% (*p* < 0.01).

### Increased expression of neutrophil Siglec-9 and levels of plasma sSiglelc-9 in COPD patients

**Figure 1:**
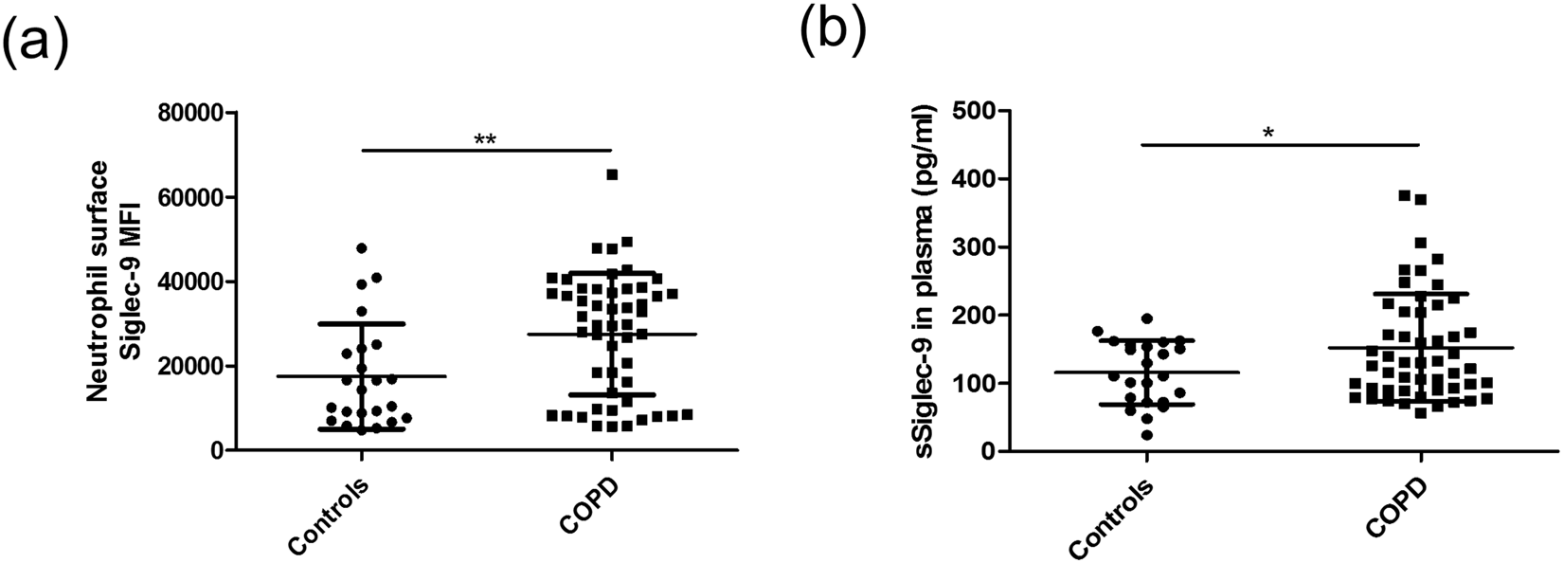
Increased expression of neutrophil Siglec-9 and plasma levels of sSiglelc-9 in COPD patients. (**a**) Siglec-9 expression on neutrophil surfaces was significantly increased in COPD patients compared with controls (*p* < 0.05) by flow cytometry analysis (MFI: mean Fluorescence Intensity). Neutrophils were isolated from the peripheral blood of controls (n = 23) and COPD patients (n = 51). (**b**) Compared with controls (n = 23), plasma sSiglelc-9 levels were significantly elevated in COPD patients (n = 51, *p* < 0.05). Data are displayed as means ± SEM. P values were calculated using Student’s t test; *p < 0.05, **p < 0.01.

Figure 1a showed that neutrophil Siglec-9 expression was significantly increased in COPD patients compared with controls (MFI 27533 ± 2020 versus 17468 ± 2598, *p* = 0.0051) by flow cytometry. The level of plasma sSiglec-9 in COPD patients was higher than controls (152.20 ± 11.03 versus 116.00 ± 9.723 pg/ml, *p* = 0.0449) (Fig. 1b).

### Increased Siglec-9 expression in alveolar neutrophil in COPD patients

**Figure 2:**
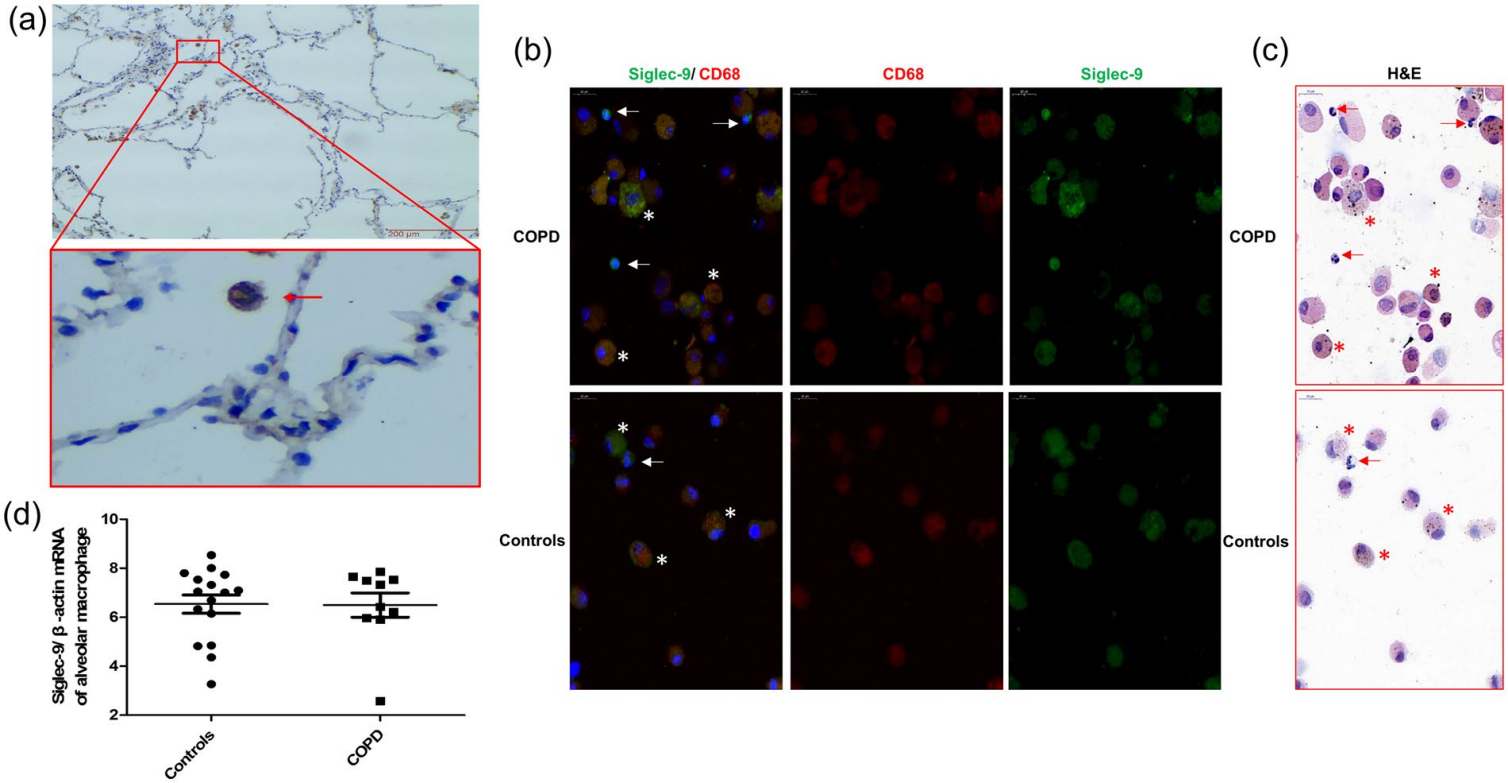
Increased Siglec-9 expression in alveolar neutrophil in COPD patients. Representative immunohistochemical images of human lung sections are shown (**a**). Arrow indicates neutrophil stained yellow brown. The red boxed area indicates a region of higher magnification. Representative images of immunofluorescence (**b**) and H&E (**c**) of alveolar neutrophil and macrophage from COPD and controls. Panel b showed double immunofluorescence staining of Siglec-9 (green) and CD68 (red). Nuclei were stained with DAPI in blue. Panel C detected alveolar cells by H&E carried out in the slides which were double staining. The arrows indicate alveolar neutrophil and asterisks points to alveolar macrophage. Siglec-9 mRNA expression in alveolar macrophage in COPD patients and controls (**d**). n = 10 for COPD patients and n = 16 for controls.

Siglec-9 expression in lung tissue was assessed by immunohistochemistry. Neutrophil and other inflammatory cells showed positive Siglec-9 staining in human alveolar spaces (Fig. 2a).

In order to determine the expression of Siglec-9 in alveolar macrophage and neutrophil, we performed dual immunofluorescence staining for Siglec-9 and CD68 (marker of macrophage) to detect macrophage and carried out H&E staining in corresponding slides to localized neutrophil. As shown in Fig. 2b and c, both alveolar macrophage and neutrophil expressed Siglec-9. Siglec-9 expression in alveolar neutrophil was markedly increased in COPD patients relative to controls. But no difference was detected in Siglec-9 expression in alveolar macrophage between two groups (Fig. 2b). To further confirm that results, the expression of Siglec-9 mRNA in alveolar macrophage was assessed by real-time PCR. Consistent with the immunofluorescence results, Siglec-9 mRNA expression was similar in COPD patients and controls (Fig. 2d).

### Neutrophil Siglec-9 expression and sSiglelc-9 levels in culture supernatant were up-regulated by CSE and LPS

**Figure 3:**
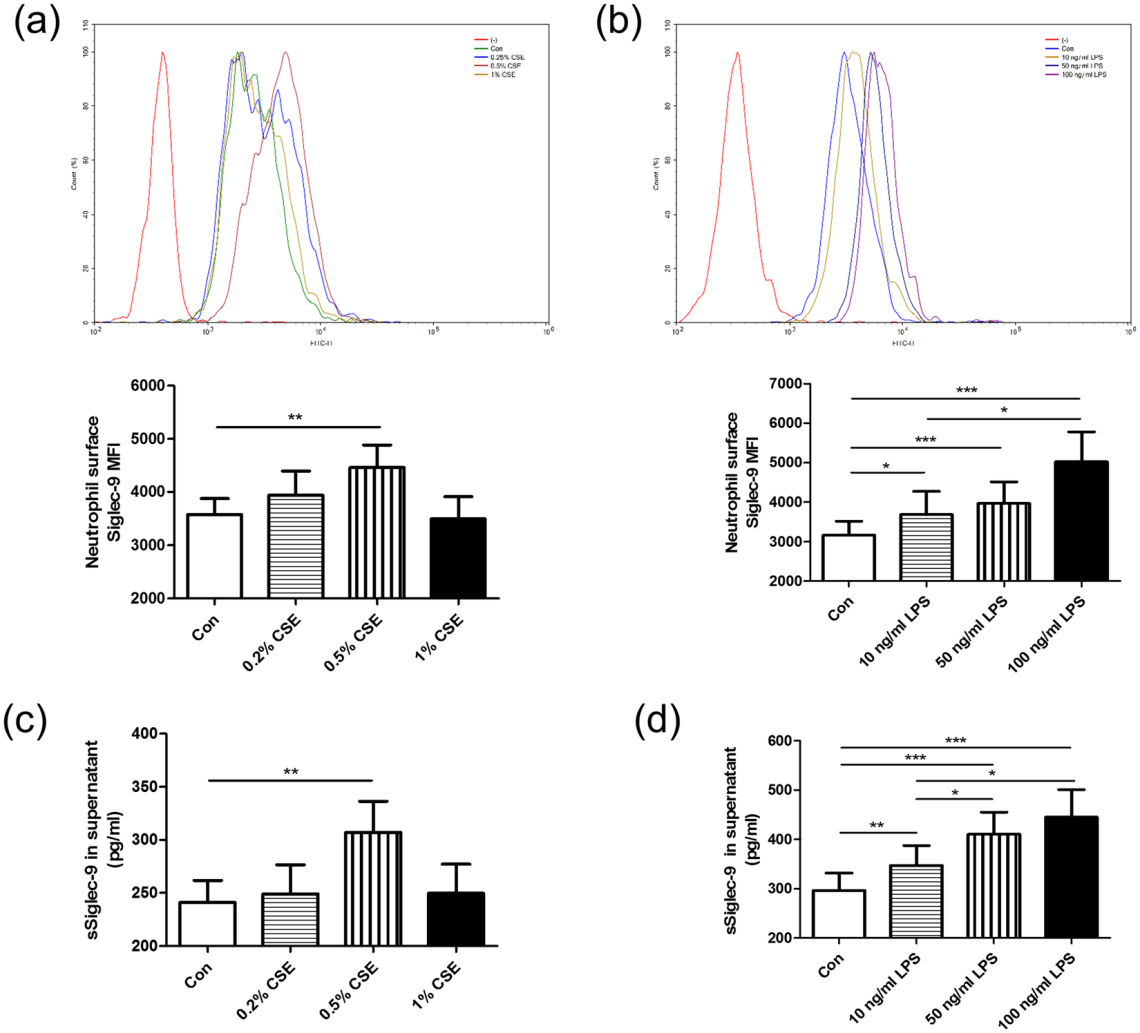
Neutrophil Siglec-9 expression and sSiglelc-9 levels in culture supernatant were up-regulated by CSE and LPS. Neutrophils were isolated from peripheral blood of controls and then incubated for 12 h in the absence or presence of CSE or LPS at different concentrations. Following culture, neutrophil Siglec-9 expression was quantified by flow cytometry analysis, and sSiglelc-9 levels in culture supernatant were determined by ELISA. (**a**,**c**) Siglec-9 and sSiglec-9 expression were up-regulated after 0.5% CSE treatment. **(b**,**d**) LPS increased Siglec-9 and sSiglec-9 expression in a concentration-dependent manner. Data are displayed as means ± SEM; n = 15. *P* values were calculated using paired t tests; *p < 0.05, **p < 0.01, ***p < 0.001.

To assess the potential effect of CSE and LPS on neutrophil Siglec-9 expression and sSiglelc-9 levels in culture supernatant, neutrophil was stimulated with various concentrations of CSE and LPS for 12 h. Only 0.5% CSE induced neutrophil Siglec-9 expression relative to control cells (MFI 4461 ± 425 versus 3575 ± 298.7, *p* = 0.0042) (Fig. 3a). Similarly, just 0.5% CSE significantly increased sSiglelc-9 levels in culture supernatant (307.3 ± 29.07 versus 241.3 ± 20.42 pg/ml, *p* = 0.0010) (Fig. 3c), while other concentrations of CSE had no effect on expression of Siglec-9. Siglec-9 expression and sSiglec-9 secretion were up-regulated after treatment with LPS in a concentration-dependent manner (Fig. 3b,d).

### Effect of cytokines on Siglec-9 and sSiglec-9 expression

**Figure 4:**
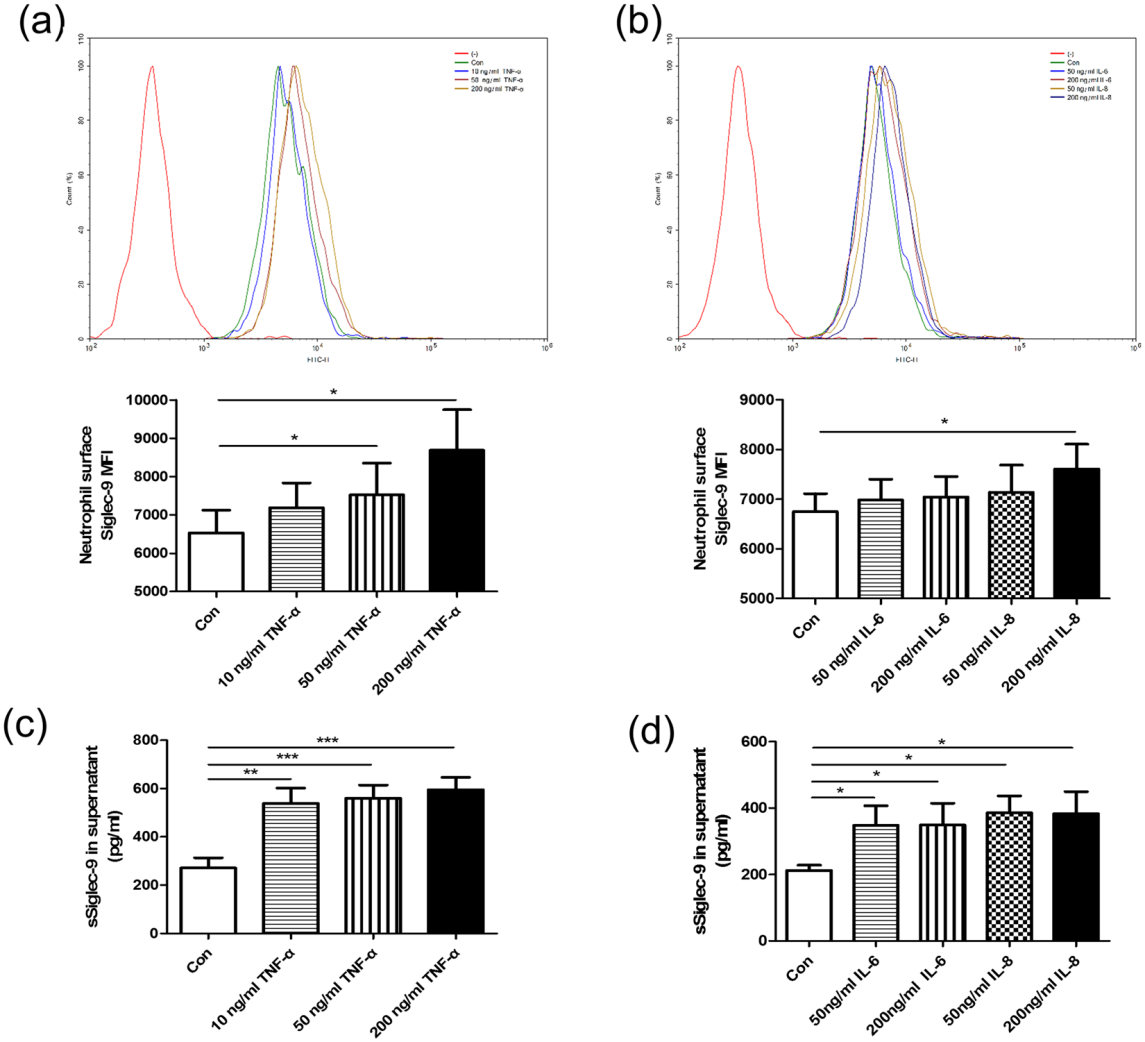
Effect of cytokines on Siglec-9 and sSiglec-9 expression. Neutrophils were isolated from peripheral blood of controls and incubated for 12 h in the absence or presence of recombinant cytokines. Following culture, neutrophil Siglec-9 expression was quantified by flow cytometry analysis, and sSiglelc-9 levels in culture supernatant was determined by ELISA. (**a**,**b**) Siglec-9 expression was up-regulated after incubation with recombinant TNF-а and IL-8. (**c**,**d**) Levels of sSiglec-9 in culture supernatant were increased after stimulation with recombinant cytokines. Data are displayed as means ± SEM; n = 15. *P* values were calculated using paired t tests; *p < 0.05, **p < 0.01, ***p < 0.001.

CSE and LPS can induce neutrophil to secret TNF-α, IL-6, and IL-8 which contribute to the development of COPD. Siglec-9 expression was remarkably up-regulated after incubation with 50 ng/ml (MFI 7519 ± 829.8 versus 6529 ± 594.7, *p* = 0.018), 200 ng/ml TNF-α (MFI 8686 ± 1058 versus 6529 ± 594.7, *p* = 0.0185), and IL-8 (MFI 7607 ± 502.7 versus 6752 ± 358.9, *p* = 0.0124) compared to controls (Fig. 4a,b), while different concentrations of IL-6 had no effect on Siglec-9 expression (Fig. 4b). TNF-α significantly induced a higher production of sSiglec-9 relative to control cells (Fig. 4c); similar results were obtained when cells were treated with IL-6 and IL-8 (Fig. 4d).

### Dexamethasone augmented neutrophil Siglec-9 expression but not sSiglec-9 levels in culture supernatant

**Figure 5:**
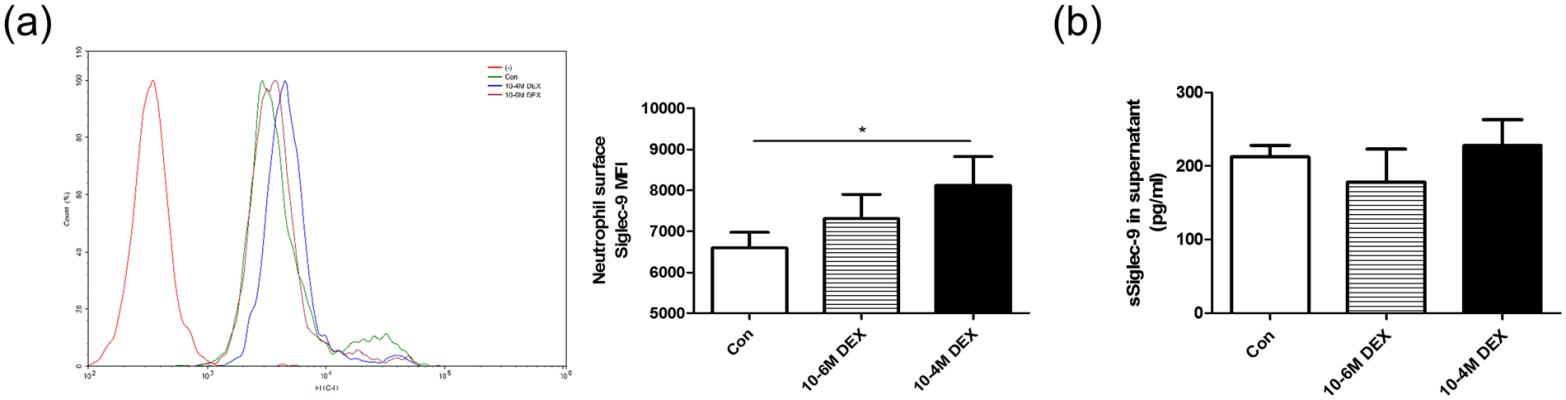
Dexamethasone augmented neutrophil Siglec-9 expression but not sSiglec-9 levels in culture supernatant. Neutrophils were isolated from peripheral blood of controls and incubated for 12 h in the absence or presence of dexamethasone. Following culture, neutrophil Siglec-9 expression was quantified by flow cytometry analysis, and sSiglelc-9 levels in culture supernatant was determined by ELISA. (**a**) 10^−4^ M DEX augmented Sigled-9 expression. (**b**) DEX had no effect on sSiglec-9 levels in culture supernatant. Data are displayed as means ± SEM, n = 15. *P* values were calculated using paired t tests; *p < 0.05.

Corticosteroids are the most widely used anti-inflammatory therapy for the treatment of COPD^22^. We found that 10^−4^ M DEX (MFI 8116 ± 705.4 versus 6594 ± 377.9, *p* = 0.0211) but not 10^−6^ M DEX augmented Sigled-9 expression but DEX did not regulate sSiglec-9 production in culture supernatant (Fig. 5a,b).

### Oxidative burst in neutrophil was increased by sSiglce-9 treatment

**Figure 6:**
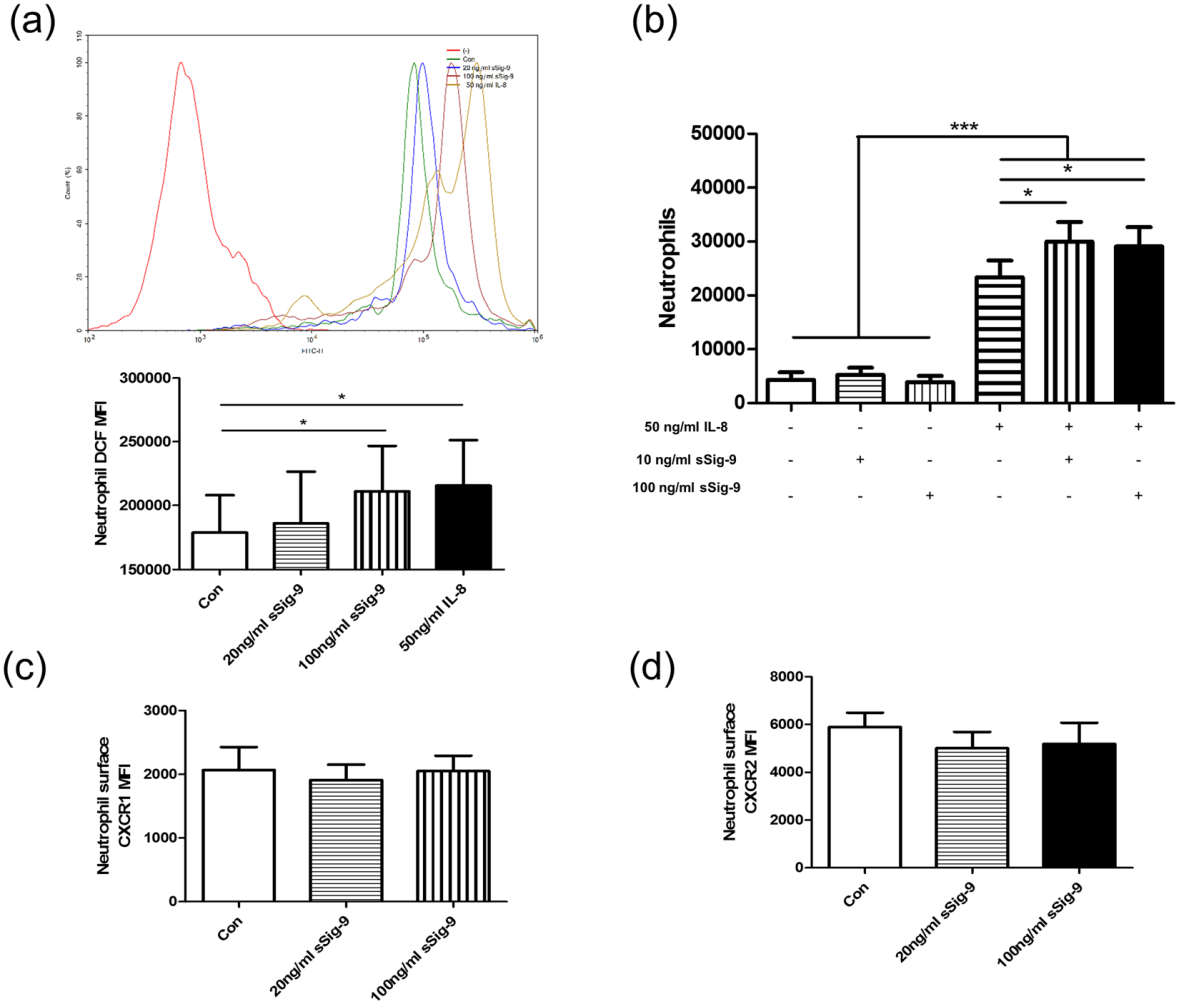
Soluble Siglce-9 increased oxidative burst in neutrophil and enhanced neutrophil chemotaxis toward IL-8 independent of CXCR1 and CXCR1 expression. Neutrophils were isolated from peripheral blood of controls and were pretreated with sSiglec-9 or recombinant IL-8 for 2 h. (**a**) 100 ng/ml sSiglce-9 and 50 ng/ml sSiglce-9 IL-8 significantly boosted levels of oxidative burst in neutrophil by flow cytometric analysis. (**b**) 1 × 10^6^ cells were added to the upper chamber and incubated in the absence or presence of IL-8 in lower compartments. Different concentrations of sSiglce-9 enhanced neutrophil migration toward IL-8. (**c** and **d**) Different concentrations of sSiglec-9 failed to affect the expression of CXCR1 and CXCR2 on neutrophil. Data are displayed as means ± SEM, n = 15. *P* values were calculated using paired t tests; *p < 0.05, **p < 0.01, ***p < 0.001.

Basal levels of intracellular ROS in neutrophil were 178915 ± 29102 (MFI), significantly lower than ones treated by 100 ng/ml sSiglec-9 (MFI 210993 ± 35751, *p* = 0.0219) and IL-8 (as positive control, MFI 215494 ± 35750, *p* = 0.0381), indicating that both aSiglec-9 and IL-8 induced oxidative stress (Fig. 6a).

### Soluble Siglec-9 enhanced neutrophil chemotaxis toward IL-8 independent of CXCR1 and CXCR2 expression

To characterize the role of sSiglec-9 in the migration of neutrophil, we examined the effect of sSiglec-9 on the random and directional (toward IL-8) neutrophil migration by using a transwell assay. As shown in Fig. 6b, neutrophil migration was significantly greater in the presence of IL-8 (*p* < 0.001). The migratory response of neutrophils toward IL-8 was enhanced by 10 ng/ml sSiglec-9 (30002 ± 3609 versus 23357 ± 3102 cells, *p* = 0.0232) and 100 ng/ml sSiglec-9 (26893 ± 3510 versus 23357 ± 3102 cells, *p* = 0.0239) (Fig. 6b); however, there were no sSiglec-9 concentration-dependent changes in IL-8-induced neutrophil migration. Besides, there was no change in the ability of neutrophil to migrate that were only pretreated with sSiglec-9.

As CXCR1 and CXCR2 play a critical role in neutrophil migration^23^, we evaluated whether their expression was changed under sSiglec-9 incubation for 2 h. Unfortunately, sSiglec-9 had no effect on their expression (Fig. 6c,d).

### CSE-induced or LPS-reduced neutrophil apoptosis were not altered by sSiglce-9

**Figure 7:**
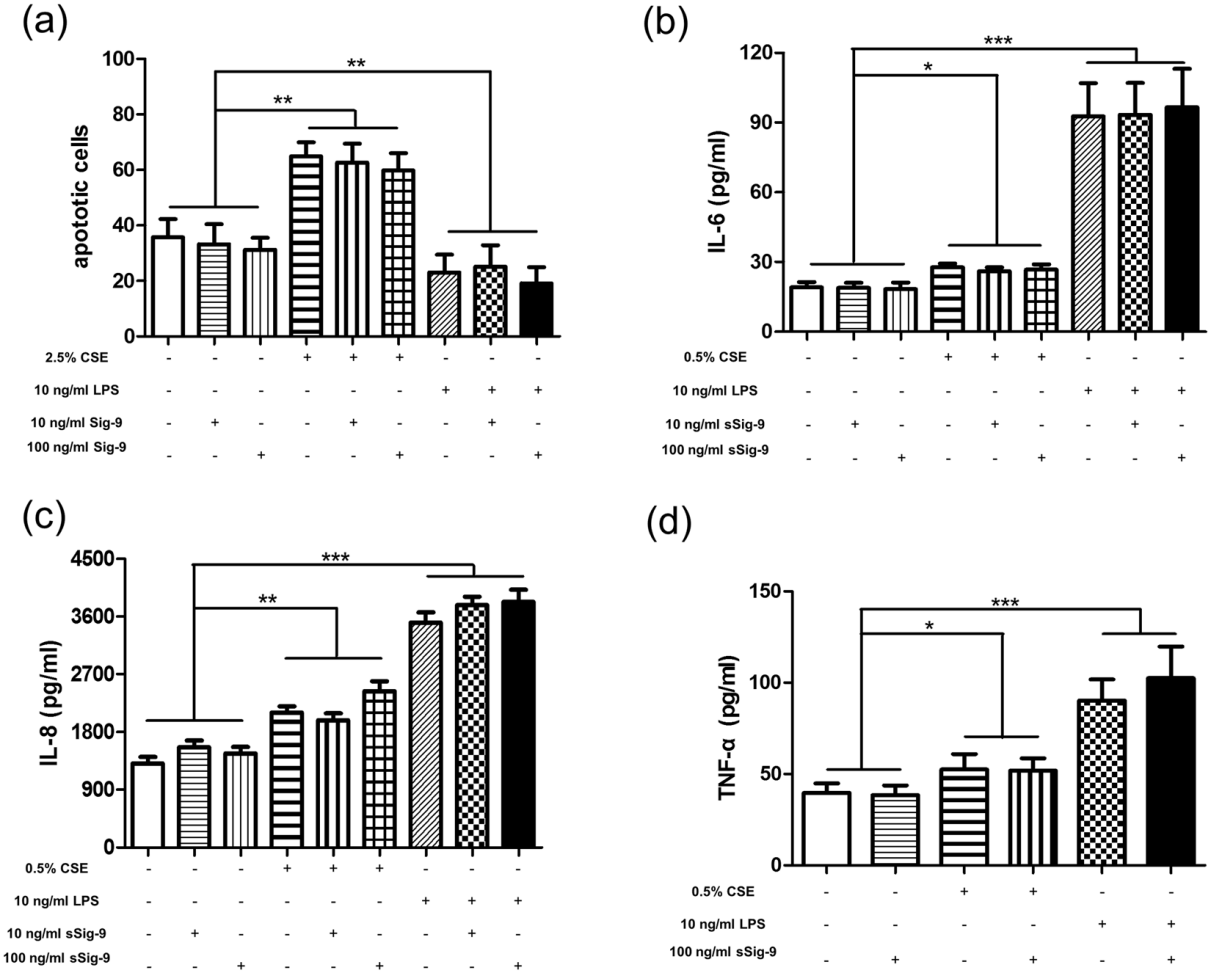
No effect of sSiglec-9 on neutrophil apoptosis and inflammatory cytokine secretion. (**a**) High levels of CSE (2.5%) induced apoptosis of neutrophils isolated from peripheral blood of controls, whereas 10 ng/ml LPS reduced apoptosis. No effect of sSiglec-9 on neutrophil apoptosis were detected through the binding of annexin V-FITC and PI. Both 0.5% CSE and 10 ng/ml LPS increased neutrophils secretion of IL-6 (**b**), IL-8 (**c**) and TNF- α (**d**) but none were affected by sSiglec-9. Data are displayed as means ± SEM, n = 15. *P* values were calculated using paired t tests; *p < 0.05, **p < 0.01, ***p < 0.001.

Compared with controls (35.71 ± 6.591), the percentage of apoptotic cells was higher in 2.5% CSE-treated cells (64.87 ± 5.062, *p* = 0.0011) but lower in LPS-treated cells (23.04 ± 6.454, *p* = 0.0035) (Fig. 7a); however, there was no effect of sSiglce-9 on neutrophil apoptosis induced by 2.5% CSE or reduced by LPS (Fig. 7a).

### Soluble Siglec-9 had no effect on neutrophil inflammatory cytokine secretion

Compared with relative controls, CSE and LPS induced neutrophil secretion of IL-6 (CSE: 27.69 ± 1.610 versus 19.06 ± 2.318 pg/ml; *p* = 0.0030; LPS: 92.64 ± 14.31 versus 19.06 ± 2.318 pg/ml, *p* = 0.0003) (Fig. 7b), IL-8 (CSE: 2103 ± 97.30 versus 1311 ± 100.7 pg/ml, *p* < 0.0001; LPS: 3505 ± 162.6 versus 1311 ± 100.7 pg/ml, *p < *0.0001) (Fig. 7c) and TNF-α (CSE: 52.61 ± 8.370 versus 39.73 ± 5.170 pg/ml, *P* = 0.0060; LPS: 90.24 ± 11.52 versus 39.73 ± 5.170 pg/ml, *p* < 0.0001) (Fig. 7d); however, sSiglec-9 did not affect neutrophil inflammatory cytokines secretion induced by CSE or LPS.

### Soluble Siglec-9 enhanced LPS-induced HBE IL-8 secretion

**Figure 8:**
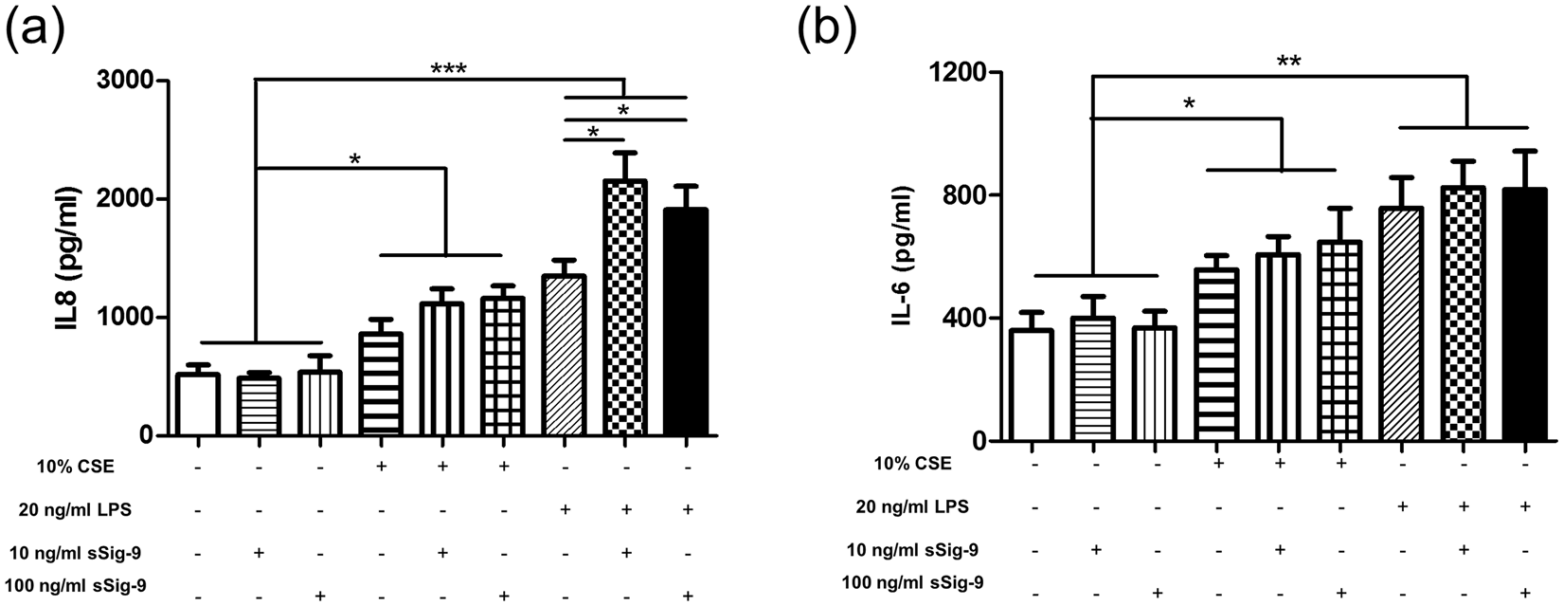
Soluble Siglce-9 enhanced LPS-induced HBE secretion of IL-8. HBE was pretreated with sSiglec-9 for 1 h and then treated with CSE and LPS. CSE and LPS increased IL-8 (**a**) and IL-6 (**b**) secretion in HBE. (**a**) Both 10 ng/ml and 100 ng/ml sSiglce-9 enhanced IL-8 secretion induced by LPS. Data are displayed as means ± SEM, n = 5. *P* values were calculated using one-way ANOVA with Newman-Keuls post hoc test; *p < 0.05, **p < 0.01, ***p < 0.001.

HBE secreted higher IL-8 and IL-6 levels than controls after stimulation with 10% CSE (IL-8: 863 ± 124.5 versus 518.9 ± 81.00 pg/ml, *p* < 0.05; IL-6: 557.8 ± 45.21 versus 359.5 ± 58.85 pg/ml, *p* < 0.05) and 20 ng/ml LPS (IL-8: 1351 ± 116.6 versus 518.9 ± 81.00 pg/ml, *p < *0.001; IL-6: 757.5 ± 99.63 versus 359.5 ± 58.85 pg/ml, *p* < 0.001) for 24 h (Fig. 8a,b). Only sSiglec-9 treatment had no effect on IL-8 secretion but pretreatment with 10 ng/ml (2153 ± 238.7 versus 1351 ± 116.6 pg/ml, *p* < 0.05) and 100 ng/ml (1812 ± 215.9 versus 1351 ± 116.6 pg/ml, *p* < 0.05) sSiglec-9 enhanced IL-8 secretion in the milieu of LPS but not CSE (Fig. 8a). Different concentrations of sSiglec-9 had no effect on CSE- and LPS-induced HBE IL-6 production (Fig. 8b).

## Discussion

In this study, we showed that neutrophil Siglec-9 expression and sSiglelc-9 levels in plasma were increased in COPD patients. *In vitro*, CSE and LPS-induced neutrophil Siglec-9 expression were likely through TNF-α and IL-8; sSiglec-9 was likewise elevated by CSE and LPS presumably via TNF-α, IL-6, and IL-8, whereas DEX only augmented neutrophil Siglec-9 expression but not sSiglec-9 levels in supernatant. As an additional functional verification, we found that sSiglce-9 increased oxidative burst in neutrophil and enhanced neutrophil chemotaxis toward IL-8. Taken together, Siglec-9 was complementarily increased to inhibit neutrophil activation in COPD patients and in cell model incubation with CSE and LPS. Instead, sSiglce-9 levels was higher in COPD patients than controls and subsequently enhanced neutrophil ROS and chemotaxis toward IL-8 probably via competitive inhibiting ligands binding to Siglec-9. Furthermore, DEX might exert an anti-inflammatory role through up-regulating Siglec-9 expression.

Neutrophil Siglec-9 expression was shown to be involved in septic shock and rheumatoid arthritis patients^10^. We found that Siglec-9 expression of alveolar and peripheral blood neutrophil were significantly increased in COPD patients. As Siglec-9 played a role in inhibiting immune response, so compensated elevation of Siglec-9 expression might exert a negative feedback loop to limit neutrophil responses. Besides, plasma sSiglelc-9 levels were significantly higher in COPD patients than controls.

Soluble Siglec-9 could prevent down-regulation of the immune responsiveness of neutrophil and provide an antibacterial benefit against Group B *Streptococcus* infection by competitively inhibiting binding of capsular polysaccharide to Siglec-9 on neutrophil surfaces^20^. Tomioka *et al.* reported that sSiglec-9 exerted an antitumor effect against Mucin1-expressing tumors by avoiding Mucin1 binding to immune cell Siglec-9 resulting in negative immunomodulation and/or by inactivating tumor-associated Mucin1 signaling^21^. Therefore, it was a reasonable assumption that sSiglec-9 might block the interaction of Siglec-9 with its ligands to disturb Siglec-9 function in COPD. This hypothesis was applied to understand why Siglec-9 and sSiglelc-9 are increased in the excessive and uncontrolled neutrophilic inflammatory airway disease^24, 25^. More specifically, Siglec-9 was of compensatory elevation in COPD and increased levels of sSiglec-9 might prevent Siglec-9 from activation via competitive inhibition of ligands binding to Siglec-9.

CS is the main risk factor for developing COPD^26^ which is accelerated through exacerbations caused by infection^27^. Consistent with previous studies showing that LPS increases Siglec-9 or Siglec-E expression^28, 29^, we found that LPS induced Siglelc-9 expression in peripheral blood neutrophil, it turned out to be truth for CSE. Besides, CSE and LPS could induce sSiglec-9 expression in the culture supernatant. Thus, CSE and LPS partially accounted for higher expression of Siglec-9 and sSiglec-9 in COPD.

CSE and LPS resulted in up-regulated production of pro-inflammatory cytokines which were involved in the pathogenesis of COPD^30^. We found that TNF-α and IL-8 increased both Siglec-9 and sSiglec-9 expression whereas IL-6 only augmented sSiglec-9 production. Therefore, TNF-α and IL-8 might attribute to CSE- and LPS-induced Siglec-9 expression. In addition, TNF-α, IL-6, and IL-8 might account for CSE- and LPS-induced sSiglec-9 production.

Leukocyte migration is critical to maintaining host defense but uncontrolled cellular infiltration into tissues can lead to chronic inflammation^31^. One key feature of COPD is excessive neutrophilic airway inflammation^24, 25^. The accumulation of neutrophil can be due to increased neutrophil recruitment^32^, delayed spontaneous apoptosis^33^, or repression of the clearance of apoptotic neutrophil by tissue macrophages^34^. IL-8 is elevated in the airways of COPD patients and was one of the chemotactic factors generated at the site of inflammation. It is a powerful mediator of neutrophil migration and activation^35, 36^. Our results found sSiglce-9 enhanced neutrophil migration toward IL-8 but had no influences on apoptosis. It was reasonable to postulate that high levels of sSiglce-9 partly contributed to excessive neutrophilic airway inflammation in COPD by elevating neutrophil recruitment. Siglec-9 and Siglec-E are important negative regulators of neutrophil and dendritic cells recruitment^12, 13^. Therefore, sSiglce-9 increased neutrophil chemotactic response probably through binding to Siglec-9 ligands to block Siglec-9 activation.

IL-8 activates neutrophil via CXCR1 and CXCR2; the latter appears to be the predominant receptor mediating its chemotactic response^23^. Circulating neutrophil CXCR1 expression is significantly higher in COPD patients than in controls^37^. We detected that neutrophil CXCR1 and CXCR2 expression were not changed after incubation with sSiglec-9 for 2 h; therefore, sSiglec-9 enhanced neutrophil chemotaxis toward IL-8 was independent of CXCR1 and CXCR2 expression. The precise mechanisms of neutrophil chemotactic response toward IL-8 requires further investigation.

Considerable evidence links COPD with increased oxidative stress^38^. Circulating neutrophil from cigarette smokers and patients with exacerbations of COPD exhibit more oxidant burden^39^, possibly due to the fact that cigarette smoke contains more than 1014 oxidants per puff^40^ and that infection and air pollutants have the potential to produce oxidative stress^41^. Although engagement of Siglec-9 or Siglec-E suppressed oxidative stress^14, 42^, we observed that sSiglce-9 significantly boosted levels of oxidative burst in neutrophil in the present study. This observation may be due to sSiglce-9 competitive inhibiting ligands of siglec-9 binding to siglec-9.

Neutrophil apoptosis is associated with inflammatory diseases and appears to be regulated by neutrophil survival cytokines, such as granulocyte/macrophage colony-stimulating factor (GM-CSF)^43^. Several *in vitro* experiments have shown that Siglec-9 engagement with antibodies causes neutrophil death, especially in some cytokine-primed cells^10, 44^. GM-CSF has been considered to be a survival factor for neutrophil but GM-CSF was showed to promote death of neutrophil in association with Siglec-9 cross-linking when neutrophil was preincubated with GM-CSF and subsequently Siglec-9 stimulated. When GM-CSF was added at the same time or after the addition of an anti-Siglec-9 antibody, the cytokines had no effect^10^. The mechanisms underlying the interplay of neutrophil survival cytokines and Siglec-9 are complex and not completely resolved. In our study, spontaneous neutrophil apoptosis was not altered by sSiglec-9 stimulation; high level CSE-induced and LPS-reduced neutrophil apoptosis were also not affected, possibly due to the interplay of neutrophil survival cytokines and Siglec-9.

Matsubara *et al*. observed only a combination of monocyte chemoattractant protein-1 and sSiglec-9, but not alone, repaired spinal cord injury by anti-inflammatory M2-like macrophage induction^45^. However, Matsumoto *et al*. reported that sSiglec-9 alone alleviated the joint inflammation and destruction in a collagen-induced arthritis by suppression of M1 proinflammatory macrophage, which led to lower levels of TNF-a and IL-6^46^. Contrary to their results, we found that sSiglec-9 had no effect on neutrophil or smooth muscle cell (data not shown) secretion of inflammatory cytokines but enhanced LPS-induced HBE secretion of IL-8 which might subsequently recruit more neutrophil into the lungs. Thus, this result highlighted that sSiglec-9 might induce a positive feedback loop to aggregate airway inflammation in COPD by enhancing IL-8 production under infection. The impacts of sSiglec-9 on cytokine secretion appear to be cell type-specific or dependent on cytokines in the circumstance.

Corticosteroids, anti-inflammatory drugs, are therapeutic agents for relieving COPD symptoms^22^. Siglec-5/14 expression is significantly higher in sputum cells from COPD patients receiving inhaled corticosteroids than those without them^16^. DEX is a synthetic corticosteroid and has been widely used for the treatment of COPD. In the current study, we found that DEX augmented neutrophil Siglec-9 expression but not sSiglec-9 levels in culture supernatant. These results indicate that DEX might exert an anti-inflammatory effect on neutrophil by up-regulating Siglec-9 expression.

In conclusion, Siglec-9 was complementarily increased to serve as a negative feedback loop to limit neutrophil activation in COPD. Soluble Siglce-9 was higher in COPD patients and subsequently enhanced neutrophil ROS and chemotaxis toward IL-8 likely via competitively inhibition of ligands binding to Siglec-9. Furthermore, DEX might exert an anti-inflammatory role through up-regulating Siglec-9 expression.

## Methods

### Subjects

A total of 51 COPD patients and 23 healthy controls were included in our study. The clinical characteristics of them are described in Supplementary Table S1. The COPD patients were recruited from outpatient of the Tongji hospital between April 2014 and April 2016. COPD was diagnosed according to the criteria established by the NHLBI/WHO Global Initiative for COPD^47^. Control subjects were selected from a pool of healthy men who visited the general health check-up center in the Tongji hospital. Subjects were excluded if they suffered from asthma, other obstructive lung diseases, tumors or a course of oral corticosteroids in the previous 4 weeks. 15 ml blood samples were collected from all participants.

Bronchoalveolar lavage fluid (BALF) was obtained from subjects who were required bronchoscopic evaluation of a solitary pulmonary nodule between September 2016 and June 2017 in Tongji hospital. A total of 10 COPD patients and 16 controls were included. The clinical characteristics of them are shown in Supplementary Table S2. Subjects with history of asthma, allergy, pneumonia, interstitial lung diseases and bronchiectasis were excluded.

Lung tissues specimens were collected during surgical resection of solitary pulmonary nodules as described previously^48^.

This study was conducted in compliance with the institutional policy regarding the protection of patients’ private information and approved by the Ethical Committee of Tongji Hospital, Huazhong University of Science and Technology (IRB ID: 20140415). Written informed consent was obtained from all participants. Sample collection and all the experimental methods in our study were carried out in accordance with the approved guidelines.

### Isolation and culture of neutrophil

Neutrophil was isolated from peripheral blood by Ficoll-Hypaque gradient centrifugation as described previously^49^. Briefly, peripheral blood was mixed with equal volume of hydroxyethyl starch 550 (TBDscience, Tianjin, China) and PBS, and allowed to sediment for 30 min at room temperature. The leukocyte containing supernatant was then carefully layered onto gradient reagent (TBDscience, Tianjin, China). After centrifugation at 800 g for 25 min, all the layers without polymorphonuclear (PMN) were removed. This method routinely yielded a purity >98% as determined by Wright-Giemsa staining. The viability of isolated cells was found to be greater than 96% with trypan blue (Sigma, St. Louis, USA) exclusion. After washing in phosphate-buffered saline solution, both cells were suspended at concentration of 5 × 106 cells/mL in 1640 RPMI medium (GIBCO Laboratories, Grand Island, NY) in 6-well plates (Nest, China).

### BALF collection and isolation of Bronchoalveolar Macrophage

During fiberoptic bronchoscopy, BALF was collected prior to any other procedures according to international guidelines^50^. The bronchoscope was wedged in the subsegmental bronchus of the middle lobe not containing any nodule. The bronchus was lavaged with 50 ml aliquots of sterile saline solution at 37 °C and then the fluid was aspirated. Two further 50 ml aliquots of saline solution were instilled and aspirated in the same way. Aliquots were transferred to 50 ml polypropylene tubes on ice.

BALF was strained through a 40 μm cell strainer (Millipore, Germany) and then centrifuged at 500 × g for 10 min at 4 °C. The cell pellet was resuspended in 2 ml RPMI 1640 and total cell viability was determined by trypan blue exclusion. Slides were prepared by cytocentrifugation at 500 × g for 5 min. slide were stained with a standard May-Grunwald Giemsa stain and a differential count was performed by examining 300 cells. Other slides were fixed by 4% formaldehyde for immunofluorescence.

Bronchoalveolar macrophage were allowed to adhere to sterile 6-well polystyrene culture plates (Costar, NY, USA) at 37 °C for 2 h. Nonadherent cells were removed by washing the monolayers with PBS, yielding monolayers that contained at least 95% macrophages by morphologic criteria. 1 ml TRIzol was added for extracting total RNA.

### Cell stimulation

To determine the siglec-9 expression on neutrophil surface, cells were stimulated with different concentration of CSE^51^, LPS (sigma-Aldrich, St. Louis, USA), TNF-α, IL-6 and IL-8 (Peprotech, Rocky Hill, UAS) for indicated time. In order to determine the effect of sSiglec-9 on neutrophil or bronchial epithelial cells (HBE) (ATCC, VA, USA) cytokines secretion, cells were pretreated with recombinant siglec-9 (sSiglec-9) (R&D Systems, Minneapolis, USA) for 1 h, and then treated with CSE or LPS for indicated time. Supernatants were stored at −80 °C for ELISA analysis, and cells were collected for detection.

### Flow cytometry

After treatment, cells were washed and resuspended in 300 μl Cell Staining Buffer (Biolegend, San Diego, USA). Cells were incubated with the appropriate primary antibodies for 30 minutes in the dark. Primary antibodies are as following: Human Siglec-9 Fluorescein (FITC)-conjugated antibody and IgG2A isotype control-FITC were obtained from R&D Systems (Minneapolis, USA); Human CXCR1 FITC-conjugated antibody, IgG2B isotype control-FITC, Human CXCR2 phycoerythrin (PE)-conjugated antibody as well as IgG1 isotype control-PE were obtained from Biolegend (San Diego, USA). Samples were washed twice in ice-cold PBS and resuspended in 300 μl Cell Staining Buffer (Biolegend, San Diego, USA), and then assessed by flow cytometry using a Becton Dickinson LSR flow cytometer^48^.

### Intracellular ROS detection

Intracellular ROS level was detected using Reactive Oxygen Species Assay Kit (Beyotime, Jiangsu, China). Neutrophil was pretreated with sSiglec-9 or recombinant IL-8 for 2 h. After treatment, cells were washed and incubated in RPMI 1640 medium with 10 uM dichlorofluorescein diacetate (DCFH-DA) for 20 min. Subsequently, the cells were washed 3 times with the RPMI 1640 medium without serum. Finally, the cells were resuspended with 300 μl PBS, and the mean fluorescent signal intensity (MFI) of dichlorofluorescein (DCF) was determining by flow cytometry.

### Assessment of apoptosis

An annexinV–fluorescein isothiocyanate (FITC) apoptosis detection kit (KeyGEN, Nanjing, China) was used for detection of neutrophil apoptosis^49^.

### Immunofluorescence and H&E staining

After rehydration in a graded alcohol series, slides of BALF were washed in TBS and heated at 100 °C for 10 min in sodium citrate buffer for epitope retrieval. Slides were incubated with blocking reagent (Dako Japan Ltd., Kyoto, Japan) for 1 h at room temperature, and then were incubated overnight with anti-CD68 antibody (Abcam, Cambridge, UK) plus anti-Siglec-9 antibody (R&D Systems, Minneapolis, USA) or matched isotype controls (Becton Dickinson) at 4 °C. After washing, samples were incubated with Alexa Fluor 594-goat anti-mouse and Alexa Fluor 488-donkey anti-goat secondary antibody (Invitrogen, California, USA) for 60 min at room temperature. Nuclei were counterstained with DAPI. Subsequently, H&E staining were carried out in the slides which were double immunostaining for Siglec-9 and CD68.

### Immunohistochemistry

The lung sections were stained with anti-Siglec-9 antibody (R&D Systems, Minneapolis, USA) using a modified method as previously described^52^. A Nikon Spot image acquisition and processing system was used for image assessment.

### RNA isolation and quantitative real-time PCR

Total RNA was extracted and real-time PCR was performed as described previously^49^. Primers were as follows: β-actin F-5′-AGAAAATCTGGCACCACACCT-3′, β-actin R-5′-GATAGCACAGCCT-GGATAGCA-3′; Siglec-9 F-5′-CCACATACCAAGAATTGCACCC-3′, Siglec-9 R-5′-ACAGAGAGCCGGTGATGTTTAT-3′.

### Neutrophil chemotaxis assays

The activity of neutrophil migration was estimated through a polycarbonate filter with 3-μm pore size in 24-well transwell chambers (Costar, NY, USA). Neutrophil was incubated with sSiglec-9 for 2 hours in RPMI 1640 medium supplemented with 2% heat-inactivated FCS. Lower compartments were filled with 500 μl RPMI 1640 medium with or without 50 ug/ml IL-8 (Peprotech, Rocky Hill, USA). Then, 200 μl neutrophil suspension (1 × 10^6^/ml) was added to upper chamber and incubated for 1 hour. Cells on the lower surface of the filters and in the lower chamber were harvested in 300 μl PBS and counted by flow cytometry acquiring events for a fixed time period of 60 seconds^53, 54^.

### Enzyme linked immunosorbent assay (ELISA)

Concentrations of IL-6, IL-8 and TNF-а in cell-free culture supernatants or sSiglec-9 in plasma were measured using DuoSet ELISA kits (R&D Systems, Minneapolis, USA) as described previously^49^. The limits of detection for the IL-6, IL-8, TNF-а and sSiglec-9 ELISA kits were 9.38, 31.3, 15.6 and 62.5 pg/mL, respectively.

### Statistical analyses

Data were presented as means ± SEM. Statistical analyses were performed using Prism 5 software (GraphPad Software Inc., San Diego, USA). Normally distributed data were analyzed using Student’s t test for two groups and one-way ANOVA with Newman-Keuls post hoc test for multiple comparisons. Data which did not meet normally distribution were evaluated by Mann–Whitney U-test. The chi-square test was used to compare proportions in two groups. The number (n) of independent experiments were indicated in each case. All data were analyzed using two–tailed tests, P < 0.05 was considered to indicate a statistically significant difference.

## Electronic supplementary material

Supplementary Information

## References

[1] Fang X, Wang X, Bai C (2011). COPD in China: the burden and importance of proper management. Chest.

[2] Vestbo J (2013). Global strategy for the diagnosis, management, and prevention of chronic obstructive pulmonary disease: GOLD executive summary. American journal of respiratory and critical care medicine.

[3] Meijer M, Rijkers GT, van Overveld FJ (2013). Neutrophils and emerging targets for treatment in chronic obstructive pulmonary disease. Expert review of clinical immunology.

[4] Barnes PJ (2008). Immunology of asthma and chronic obstructive pulmonary disease. Nature reviews. Immunology.

[5] Oudijk EJ, Lammers JW, Koenderman L (2003). Systemic inflammation in chronic obstructive pulmonary disease. The European respiratory journal. Supplement.

[6] Ekberg-Jansson A (2001). Neutrophil-associated activation markers in healthy smokers relates to a fall in DL(CO) and to emphysematous changes on high resolution CT. Respiratory medicine.

[7] Rahman I, Adcock IM (2006). Oxidative stress and redox regulation of lung inflammation in COPD. The European respiratory journal.

[8] Crocker PR, Paulson JC, Varki A (2007). Siglecs and their roles in the immune system. Nature reviews. Immunology.

[9] Crocker PR (2005). Siglecs in innate immunity. Current opinion in pharmacology.

[10] von Gunten S (2005). Siglec-9 transduces apoptotic and nonapoptotic death signals into neutrophils depending on the proinflammatory cytokine environment. Blood.

[11] Avril T, Floyd H, Lopez F, Vivier E, Crocker PR (2004). The membrane-proximal immunoreceptor tyrosine-based inhibitory motif is critical for the inhibitory signaling mediated by Siglecs-7 and −9, CD33-related Siglecs expressed on human monocytes and NK cells. Journal of immunology.

[12] McMillan SJ, Sharma RS, Richards HE, Hegde V, Crocker PR (2014). Siglec-E promotes beta2-integrin-dependent NADPH oxidase activation to suppress neutrophil recruitment to the lung. The Journal of biological chemistry.

[13] McMillan SJ (2013). Siglec-E is a negative regulator of acute pulmonary neutrophil inflammation and suppresses CD11b beta2-integrin-dependent signaling. Blood.

[14] Schwarz, F. *et al*. Siglec receptors impact mammalian lifespan by modulating oxidative stress. *eLife* **4** (2015). 10.7554/eLife.06184 PMC4384638 25846707

[15] Angata T (2013). Loss of Siglec-14 reduces the risk of chronic obstructive pulmonary disease exacerbation. Cellular and molecular life sciences: CMLS.

[16] Wielgat P (2015). Inhaled corticosteroids increase siglec-5/14 expression in sputum cells of COPD patients. Advances in experimental medicine and biology.

[17] Ishii, T. *et al*. Influence of SIGLEC9 polymorphisms on COPD phenotypes including exacerbation frequency. *Respirology* (2016). 10.1111/resp.12952 27878892

[18] Carlin AF (2009). Molecular mimicry of host sialylated glycans allows a bacterial pathogen to engage neutrophil Siglec-9 and dampen the innate immune response. Blood.

[19] Belisle JA (2010). Identification of Siglec-9 as the receptor for MUC16 on human NK cells, B cells, and monocytes. Molecular cancer.

[20] Saito M (2016). A soluble form of Siglec-9 provides a resistance against Group B Streptococcus (GBS) infection in transgenic mice. Microbial pathogenesis.

[21] Tomioka Y (2014). A soluble form of Siglec-9 provides an antitumor benefit against mammary tumor cells expressing MUC1 in transgenic mice. Biochemical and Biophysical Research Communications.

[22] Hele DJ, Belvisi MG (2003). Novel therapies for the treatment of inflammatory airway disease. Expert opinion on investigational drugs.

[23] Wuyts A (1998). Differential usage of the CXC chemokine receptors 1 and 2 by interleukin-8, granulocyte chemotactic protein-2 and epithelial-cell-derived neutrophil attractant-78. European journal of biochemistry.

[24] Stanescu D (1996). Airways obstruction, chronic expectoration, and rapid decline of FEV1 in smokers are associated with increased levels of sputum neutrophils. Thorax.

[25] Lapperre TS (2007). Small airways dysfunction and neutrophilic inflammation in bronchial biopsies and BAL in COPD. Chest.

[26] Cosio MG, Saetta M, Agusti A (2009). Immunologic aspects of chronic obstructive pulmonary disease. The New England journal of medicine.

[27] Donaldson GC, Seemungal TA, Bhowmik A, Wedzicha JA (2002). Relationship between exacerbation frequency and lung function decline in chronic obstructive pulmonary disease. Thorax.

[28] Ando M, Tu W, Nishijima K-i, Iijima S (2008). Siglec-9 enhances IL-10 production in macrophages via tyrosine-based motifs. Biochemical and Biophysical Research Communications.

[29] Boyd CR (2009). Siglec-E is up-regulated and phosphorylated following lipopolysaccharide stimulation in order to limit TLR-driven cytokine production. Journal of immunology.

[30] Caramori G, Adcock IM, Di Stefano A, Chung KF (2014). Cytokine inhibition in the treatment of COPD. International journal of chronic obstructive pulmonary disease.

[31] Geng JG (2001). Directional migration of leukocytes: their pathological roles in inflammation and strategies for development of anti-inflammatory therapies. Cell research.

[32] Sapey E (2011). Behavioral and structural differences in migrating peripheral neutrophils from patients with chronic obstructive pulmonary disease. American journal of respiratory and critical care medicine.

[33] Pletz MW, Ioanas M, de Roux A, Burkhardt O, Lode H (2004). Reduced spontaneous apoptosis in peripheral blood neutrophils during exacerbation of COPD. The European respiratory journal.

[34] Minematsu N, Blumental-Perry A, Shapiro SD (2011). Cigarette smoke inhibits engulfment of apoptotic cells by macrophages through inhibition of actin rearrangement. American journal of respiratory cell and molecular biology.

[35] Yamamoto C (1997). Airway inflammation in COPD assessed by sputum levels of interleukin-8. Chest.

[36] Schulz C (2003). Expression and release of interleukin-8 by human bronchial epithelial cells from patients with chronic obstructive pulmonary disease, smokers, and never-smokers. Respiration; international review of thoracic diseases.

[37] Yamagata T (2007). Overexpression of CD-11b and CXCR1 on circulating neutrophils: its possible role in COPD. Chest.

[38] McCusker K (1992). Mechanisms of respiratory tissue injury from cigarette smoking. The American journal of medicine.

[39] Rahman I, Morrison D, Donaldson K, MacNee W (1996). Systemic oxidative stress in asthma, COPD, and smokers. American journal of respiratory and critical care medicine.

[40] Pryor WA, Stone K (1993). Oxidants in cigarette smoke. Radicals, hydrogen peroxide, peroxynitrate, and peroxynitrite. Ann N Y Acad Sci.

[41] Repine JE, Bast A, Lankhorst I (1997). Oxidative stress in chronic obstructive pulmonary disease. Oxidative Stress Study Group. American journal of respiratory and critical care medicine.

[42] Secundino, I. *et al*. Host and pathogen hyaluronan signal through human siglec-9 to suppress neutrophil activation. Journal of molecular medicine (2015). 10.1007/s00109-015-1341-8 PMC4766071 26411873

[43] Colotta F, Re F, Polentarutti N, Sozzani S, Mantovani A (1992). Modulation of granulocyte survival and programmed cell death by cytokines and bacterial products. Blood.

[44] von Gunten S (2006). Immunologic and functional evidence for anti-Siglec-9 autoantibodies in intravenous immunoglobulin preparations. Blood.

[45] Matsubara K (2015). Secreted ectodomain of sialic acid-binding Ig-like lectin-9 and monocyte chemoattractant protein-1 promote recovery after rat spinal cord injury by altering macrophage polarity. The. Journal of neuroscience: the official journal of the Society for Neuroscience.

[46] Matsumoto T (2016). Soluble Siglec-9 suppresses arthritis in a collagen-induced arthritis mouse model and inhibits M1 activation of RAW264.7 macrophages. Arthritis research & therapy.

[47] Rabe KF (2007). Global strategy for the diagnosis, management, and prevention of chronic obstructive pulmonary disease: GOLD executive summary. American journal of respiratory and critical care medicine.

[48] Xia J (2015). Increased IL-33 expression in chronic obstructive pulmonary disease. American journal of physiology. Lung cellular and molecular physiology.

[49] Wang M (2016). Impaired anti-inflammatory action of glucocorticoid in neutrophil from patients with steroid-resistant asthma. Respiratory research.

[50] Meyer KC (2012). An official American Thoracic Society clinical practice guideline: the clinical utility of bronchoalveolar lavage cellular analysis in interstitial lung disease. American journal of respiratory and critical care medicine.

[51] Xu Y (2013). Cigarette smoke (CS) and nicotine delay neutrophil spontaneous death via suppressing production of diphosphoinositol pentakisphosphate. Proceedings of the National Academy of Sciences of the United States of America.

[52] Giaid A (1993). Expression of endothelin-1 in lungs of patients with cryptogenic fibrosing alveolitis. Lancet.

[53] Rios-Santos F (2007). Down-regulation of CXCR2 on neutrophils in severe sepsis is mediated by inducible nitric oxide synthase-derived nitric oxide. American journal of respiratory and critical care medicine.

[54] Scandella E (2002). Prostaglandin E2 is a key factor for CCR7 surface expression and migration of monocyte-derived dendritic cells. Blood.

