# Supplementary material for: Increased expression of Siglec-9 in chronic obstructive pulmonary disease
Source: Sci Rep. 2017 Aug 31;7:10116. doi: 10.1038/s41598-017-09120-5 (PMC5579055; doi:10.1038/s41598-017-09120-5)
Supplement: Supplementary file 1 — Supplementary Information [file 41598_2017_9120_MOESM1_ESM.docx]

**Increased expression of Siglec-9 in** **chronic obstructive pulmonary disease**

Zhilin Zeng^1,2^, Miao Li^1^, Meijia Wang^1^, Xiaomei Wu^1^, Qinghai Li^1^, Qin Ning^2^, Jianping Zhao^1^, Yongjian Xu^1^, Jungang Xie^1*^

^1^ Department of Respiratory and Critical Care Medicine, National Clinical Research Center of Respiratory Disease, Tongji Hospital, Tongji Medical College, Huazhong University of Science and Technology, Wuhan 430030, China.

^2^ Department of Infectious Disease, Institute of Infectious Disease, Tongji Hospital of Tongji Medical College, Huazhong University of Science and Technology, Wuhan 430030, China.

**Supplementary Table S1.** **Characteristics of subjects providing peripheral blood**

|  | Controls | COPD |
| --- | --- | --- |
| N | 23 | 51 |
| Age(years) | 61.50±1.69 | 64.20±1.01 |
| Sex(M:F) | 22:1 | 50:1 |
| Smoking(packs/year) | 40.89±4.63 | 49.96±3.24 |
| FEV_1_(L) | 2.80±0.10 | 1.44±0.07*** |
| FEV_1_ % predicted | 93.82±2.21 | 51.83±2.25*** |
| FEV_1_/FVC | 78.76±1.05 | 48.62±1.47*** |

Data presented as number or means ± SEM. FEV_1_=forced expiratory volume in I second; FVC=forced vital capacity packets/year=1 year of smoking 20 cigarettes per day. The chi-square test was used to compare sex ratios in two groups, Student’s t test was used to compare the other characteristics. ****p*<0.001 versus Controls.

**Supplementary Table S2.** **Characteristics of subjects providing BALF**

|  | Controls | COPD |
| --- | --- | --- |
| N | 16 | 10 |
| Age(years) | 57.07±2.38 | 61.00±2.55 |
| Sex(M:F) | 15:1 | 9:1 |
| Smoking(packs/year) | 40.40±7.08 | 38.57±4.04 |
| FEV_1_(L) | 3.05±0.20 | 2.11±0.27* |
| FEV_1_ % predicted | 92.34±2.23 | 67.71±6.12** |
| FEV_1_/FVC | 76.35±1.27 | 58.22±5.37** |

Data presented as number or means ± SEM. FEV_1_=forced expiratory volume in I second; FVC=forced vital capacity; packets/year=1 year of smoking 20 cigarettes per day. The chi-square test was used to compare sex ratios in two groups, Student’s t test was used to compare the other characteristics. **p*<0.05 and ***p*<0.01.
